# The Hyperproliferation Mechanism of Cholesteatoma Based on Proteomics: SNCA Promotes Autophagy-Mediated Cell Proliferation Through the PI3K/AKT/CyclinD1 Signaling Pathway

**DOI:** 10.1016/j.mcpro.2023.100628

**Published:** 2023-08-01

**Authors:** Miao Gao, Heng Xiao, Yonglan Liang, Huimin Cai, Xiaojing Guo, Jianwei Lin, Suling Zhuang, Jianhua Xu, Shengnan Ye

**Affiliations:** 1 Department of Otorhinolaryngology Head and Neck Surgery, Fujian Institute of Otorhinolaryngology, the First Affiliated Hospital, Fujian Medical University, Fuzhou, China; 2 Department of Otorhinolaryngology Head and Neck Surgery, National Regional Medical Center, Binhai Campus of the First Affiliated Hospital, Fujian Medical University, Fuzhou, China; 3 Department of Pharmacology, School of Pharmacy, Fujian Provincial Key Laboratory of Natural Medicine Pharmacology, Fujian Medical University, Fuzhou, China

**Keywords:** proteomics, cholesteatoma, alpha-synuclein, autophagy, proliferation

## Abstract

Cholesteatoma is a chronic inflammatory ear disease with abnormal keratinized epithelium proliferation and tissue damage. However, the mechanism of keratinized epithelium hyperproliferation in cholesteatoma remains unknown. Hence, our study sought to shed light on mechanisms affecting the pathology and development of cholesteatoma, which could help develop adjunctive treatments. To investigate molecular changes in cholesteatoma pathogenesis, we analyzed clinical cholesteatoma specimens and paired ear canal skin with mass spectrometry–based proteomics and bioinformatics. From our screen, alpha-synuclein (SNCA) was overexpressed in middle ear cholesteatoma and might be a key hub protein associated with inflammation, proliferation, and autophagy in cholesteatoma. SNCA was more sensitive to lipopolysaccharide-induced inflammation, and autophagy marker increase was accompanied by autophagy activation in middle ear cholesteatoma tissues. Overexpression of SNCA activated autophagy and promoted cell proliferation and migration, especially under lipopolysaccharide inflammatory stimulation. Moreover, inhibiting autophagy impaired SNCA-mediated keratinocyte proliferation and corresponded with inhibition of the PI3K/AKT/CyclinD1 pathways. Also, 740Y-P, a PI3K activator reversed the suppression of autophagy and PI3K signaling by siATG5 in SNCA-overexpressing cells, which restored proliferative activity. Besides, knockdown of SNCA in RHEK-1 and HaCaT cells or knockdown of PI3K in RHEK-1 and HaCaT cells overexpressing SNCA both resulted in attenuated cell proliferation. Our studies indicated that SNCA overexpression in cholesteatoma might maintain the proliferative ability of cholesteatoma keratinocytes by promoting autophagy under inflammatory conditions. This suggests that dual inhibition of SNCA and autophagy may be a promising new target for treating cholesteatoma.

## Highlights

• Autophagy was more active in cholesteatoma than normal auditory canal skin.
• SNCA interacted with autophagy and inflammation-related genes in cholesteatoma.
• SNCA promoted keratinocytes proliferation and autophagy, especially in inflammation.
• SNCA may be the key pathogenic protein of cholesteatoma proliferation phenotype.

## Graphical Abstract

**Figure:**
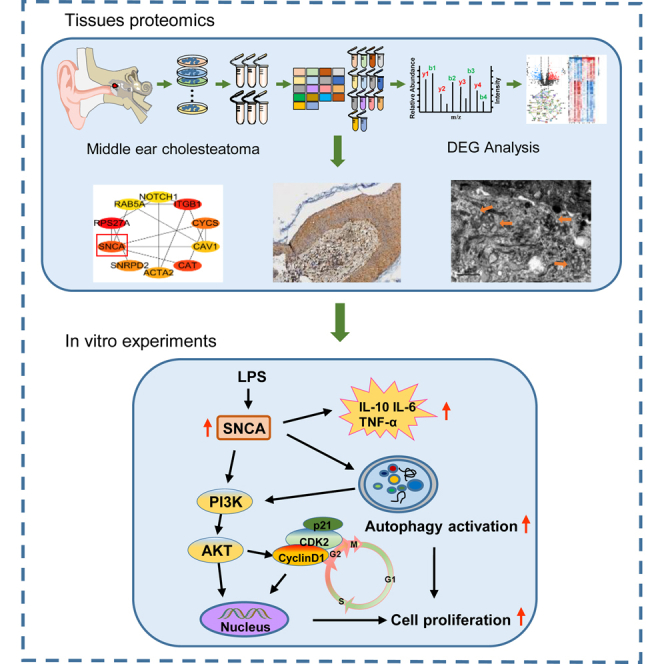

## In Brief

We analyzed clinical cholesteatoma specimens and paired ear canal skin with MS-based proteomics and bioinformatics, revealed SNCA was a key hub protein associated with inflammation, proliferation, and autophagy in cholesteatoma. Overexpression of SNCA activated autophagy and promoted cell proliferation and migration, especially under inflammatory stimulation, in part *via* PI3K/AKT/CyclinD1 signaling pathway.

Cholesteatoma is a chronic inflammatory ear disease characterized by accumulating keratinizing squamous epithelium and erosion of ossicles or temporal bone that often lead to hearing loss, vestibular dysfunction, facial nerve palsy, or intracranial complications (1, 2). Complete surgical excision is the only effective treatment of cholesteatoma, but surgery cannot prevent recurrence (3). Therefore, exploring potential molecular mechanisms of cholesteatoma development and discovering noninvasive and effective prevention or treatment approaches are particularly important.

Different theories exist concerning the pathogenesis of cholesteatoma (4, 5). These theories are mainly based on keratinizing epithelium relocalization through the tympanic membrane into the middle ear. This process involves differentiation and abnormal proliferation of keratinizing epithelial cells due to inflammation (6). Numerous studies demonstrated that cholesteatoma keratinocytes proliferate, differentiate, and migrate under inflammatory pressure (6, 7, 8). However, the mechanism for keratinocyte hyperproliferation remains unclear.

The development of mass spectrometry (MS) technology allowed for the study of disease mechanisms using MS-based proteomics, which improved our understanding of diseases at the molecular level (9). To investigate cholesteatoma pathogenesis, eight clinical specimens of cholesteatoma and paired ear canal skin were analyzed with MS-based proteomics and bioinformatics. We found that SNCA, namely, alpha-synuclein (a-Syn), was highly expressed in cholesteatoma along with upregulated inflammation, autophagy, and proliferation-related proteins. Moreover, autophagy was more active in cholesteatoma than its paired ear canal skin. In this study, we hypothesize that elevated SNCA expression promotes cholesteatoma keratinocyte proliferation by inducing autophagy in inflammatory conditions.

Autophagy is critical for lysosomal degradation of pathogens, dysfunctional or damaged organelles or proteins, and foreign bodies (10). It is essential for tissue development and differentiation (11). Autophagy can help maintain metabolism and subsequent proliferation and growth of tumors (12). Ho *et al*. and Li *et al*. found that autophagy-related proteins such as LC3 and Beclin-1 were abnormally expressed in middle ear cholestatoma (13, 14). The role of autophagy in the pathogenesis of middle ear cholesteatoma (MEC) is not known. Furthermore, it remains to be investigated whether autophagy activation in MEC tissues correlates with the adaptation and maintenance of high proliferative activity of keratinocytes in the context of chronic inflammatory stress.

*SNCA*, located in chromosome 4 (15), belongs to the synuclein family, which includes beta-synuclein and gamma-synuclein. Evidence suggests that SNCA may also induce productive autophagy in neurons (16, 17), impair early stages of autophagosome formation (18, 19), and enhance autophagic flux (20). *SNCA* has been regarded as an autophagy-related gene that influences radiotherapeutic responses in non–small cell lung cancer (21). Another study showed SNCA could be an autophagy-related marker to monitor the autophagy level of T cells in peripheral blood lymphocytes (22). However, there is little information about the specific function of SNCA in cholesteatoma pathology. Therefore, it is reasonable to propose that SNCA could regulate autophagy and epithelial proliferation in cholesteatoma.

A model of cholesteatoma epithelial cells was developed using SNCA-overexpressing keratinocyte cell lines and lipopolysaccharide (LPS) stimulation to study the possible role of SNCA in MEC. This model can be used to determine the relationship between autophagy activation and keratinocyte proliferation under chronic inflammatory stress. The study proposes a new mechanism for epidermal keratinocyte proliferation under inflammatory stress in MEC.

## Experimental Procedures

### Experimental Design and Statistical Rational

The purpose of this study was to identify markers associated with cholesteatoma pathogenesis in patients with acquired cholesteatoma. During the surgical procedure, MEC epithelium tissues and paired normal auditory canal skin (ACS) tissues were collected. The normal ACSs served as the control group. Eight pairs of samples were prepared and stored in liquid nitrogen for MS detection, ensuring biological replicates for reliable and consistent quantitative proteomic analysis. In addition, 12 pairs of samples were collected for immunohistochemical staining. In order to validate the autophagy process of cholesteatoma identified by MS, an additional four pairs of cholesteatoma tissues collected under the same standard were used for Western blot analysis and three pairs were used for electron microscopic observation. The study included patients aged between 15 and 74 years who had no history of previous surgery for the affected ear and no malignancy or autoimmune disease. The study was performed according to the principles of the Declaration of Helsinki and approved by the Ethics Committee of the First Affiliated Hospital of Fujian Medical University (IEC-FOM-013-2.0). Written informed consent was obtained from all patients. Data were analyzed by two-tailed paired Student's *t* test, and statistical significance was defined as *p* < 0.05.

### Immunohistochemistry and Transmission Electron Microscopy

For immunohistochemistry (IHC), tissues were fixed in 10% neutral-buffered formalin for 24 h and embedded in paraffin. Then, hematoxylin and eosin (H&E) staining and IHC were performed. Briefly, the tissue sections (5 μm thick) were deparaffinized and rehydrated, and endogenous peroxidase was quenched by 3% hydrogen peroxide solution for 10 min. The tissue slides were then washed with phosphate-buffered saline with Triton-X100 (PBST), then incubated with a monoclonal antibody against SNCA (1:200, ABclonal Technology) overnight at 4 °C. The slides were washed again in PBST and carefully incubated with Elivision super HRP (Mouse/Rabbit) IHC Kit (Maxim) following the manufacturer’s instructions. Diaminobenzidine (DAB kit, Maxim) was used to visualize labeled proteins, and the sections were counterstained with hematoxylin. Finally, images were captured *via* light microscopy (Nikon, amplification:200). Integrated optical density and positive staining area ratio were assessed as semiquantitative measures of SNCA expression using Image Pro Plus 6.0 software (Media Cybernetics Inc). For transmission electron microscopy, the tissue specimens were sliced into 1 × 1 × 3 mm^3^ blocks and fixed with 3% glutaraldehyde solution overnight at 4 °C. The samples were then fixed, dehydrated, embedded, sectioned at 90 nm, stained, and observed under a transmission electron microscope (Tecnai G2 Spirit Bio TWIN, FEI).

### Proteins Digestion and Tandem Mass Tag Labeling

Samples were taken from −80 °C storage, immersed in liquid nitrogen, and ground into fine powder. The processed samples were then sonicated on ice in lysis buffer (8 M urea, 1% protease inhibitor cocktail, 50 μM deubiquitinase inhibitor PR-619, 3 μM trichostatin A, and 50 mM nicotinamide). The remaining cell debris was removed by centrifugation at 4 °C (12,000*g*, 10 min). The supernatant was collected and transferred into a clean tube, and the protein concentration was measured with a BCA Protein Assay kit. Proteins were digested at 4 °C overnight with trypsin (Promega) at 1: 50 trypsin-to-protein mass ratio in 200 mM triethyl-ammonium bicarbonate. The protein solution was mixed with 5 mM DL-dithiothreitol (DTT, Sigma), incubated at 56 °C for 30 min, and subsequently alkylated with 11 mM iodoacetamide for 15 min at room temperature in the dark. A Phenomenex Strata X C18 system desalted and dried tryptic peptides using vacuum centrifugation. The peptides were dissolved in 0.5 M triethyl-ammonium bicarbonate and labeled with tandem mass tag (TMT) isobaric tags, following the manufacturer's instructions. Sample labeling was as follows: s2, 126; c2, 127N; s4, 127C; c4, 128N; s6, 128C; c6, 129N; s7, 129C; c7, 130N; s8,130C; c8, 131N; s1,131C; c1,132N; s3,132C; c3,133N; s5,133C; and c5,134N. The labeled peptides were mixed, desalted, and vacuum dried.

### HPLC Fractionation and Liquid Chromatography–Tandem Mass Spectrometry Analysis

Using an Agilent 300Extend C-18 column and a high-pH reversed-phase HPLC, tryptic peptides were fractionated into 60 fractions (5-μm particles, 4.6 mm ID, 250 mm length) over 60 min. The 60 fractions were combined into 12 fractions and dried by vacuum centrifugation for further processing.

The tryptic peptides were dissolved with solvent A (0.1% formic acid and 2% acetonitrile) and separated on an EASY-nLC 1200 (Thermo Fisher Scientific) ultraperformance liquid chromatography system using home-made reversed-phase analytical column (25 cm length, 100 μm i.d.), packed with 1.9 μm/120 Å ReproSil-PurC18 resins (Dr. Maisch GmbH, Ammerbuch, Germany).

The parameters were set as 6 to 22% solvent B (0.1% formic acid in 90% acetonitrile) for 38 min, 22 to 32% for 4 min, and increased to 80% for 4 min. The next step was maintenance at 80% for the last 4 min, all at a constant flow rate of 500 nl/min. The peptides were subjected to a nanoelectrospray ionization source and analyzed by tandem mass spectrometry (MS/MS) *via* a Q Exactive HF-X mass spectrometer (Thermo Fisher Scientific). Electrospray voltage was set at 2.1 kV, and peptides were detected with MS range 350 to 1500 *m/z* using an Orbitrap with 120,000 resolution. The MS/MS scan was set at 100 *m/z* and 45,000 resolution. The mass spectrometer was operated under data-dependent methods. With a normalized collision energy of 28%, the top 20 precursor ions from the MS full-scan were fragmented by higher-energy collisional dissociation. The MS/MS scans parameter settings were as follows: threshold ion count = 8.3 × 10^4^, AGC target value = 3 × 10^6^, and maximum ion injection time = 50 ms.

### Experimental Data Analysis

The secondary mass spectrum data were searched against a FASTA file containing human protein sequences and common contaminants (20,395 sequences, download December 2020 from UniProt database) using Proteome Discoverer (v2.4.1.15). The search parameters were configured as below: full enzyme digestion using trypsin with a maximum of two missed cleavages allowed; a requirement of at least six amino acid residues per peptide, and a maximum of three allowed peptide modifications. The peptide precursor mass tolerance was set to 10 ppm, and the fragment mass tolerance was set to 0.02 Da. Fixed modifications included carbamidomethylation (cysteine), TMTpro (K), and TMTpro (peptide N Terminus), while variable modifications included Acetyl (protein N Terminus), Deamidated (NQ), and Oxidation (methionine). Quantification was performed using the TMTpro-16plex method. The maximum false discovery rate for protein, peptide, and PSM identification was controlled at 1%. For quantitative analysis, the raw LC-MS datasets were converted into matrices containing peptide abundance across samples. The peptide abundances across all samples were subsequently centralized and transformed into relative quantification values within each sample. Then, relative peptide intensity was further normalized using the median, and the relative expression of a protein was calculated by the intensity median of its corresponding unique peptides. A ratio threshold at > 1.3 was set for protein upregulation and <0.76 (1/1.3) was set for protein downregulation.

### Bioinformatics Analysis

A protein–protein interaction network was constructed using the STRING database (v.11.5). The minimum required interaction score was 0.400. Cytoscape (v.3.8.0) software was applied to visualize the prediction network obtained from the STRING database. Gene set enrichment analysis was performed to cluster significant gene sets. Gene ontology analysis of differentially expressed proteins (DEPs) was performed using the DAVID Bioinformatics Resources 2021. Hierarchical clustering and volcano pot were drawn using the R package ggplot2.

### Cell Line and Cell Culture Conditions

Immortal human keratinocyte HaCaT cells were purchased from Procell, and the immortalized keratinocyte cell line, RHEK-1, was a gift from Dr J. Rhim (Center for Prostate Disease Research, Rockville, MD; Rhim *et al*., 1986) (23). HaCaT cells and RHEK-1 cells were cultured in high-glucose Dulbecco’s minimum essential medium and Eagle’s minimal essential medium (Hyclone), respectively, supplemented with 10% fetal bovine serum (FBS, Gibco) and 1% penicillin-streptomycin (Solarbio Science & Technology Co, Ltd). All cell lines were incubated and cultured at 37 °C and 5% CO_2_. Before our experiments, the cells were seeded in plates and allowed to adhere for 24 h.

### Lentivirus Infection and siRNA Transfection

Lentiviral constructs for SNCA overexpression (Ubi-MCS-3FLAG-SV40-puro) and an empty (control) vector was used. mRFP-GFP-LC3 and GFP-LC3 constructs were purchased from Genechem. HaCaT and RHEK-1 cells were treated with or without chloroquine (20 nM) following coinfection with lentiviruses expressing mRFP-GFP-LC3 and SNCA overexpression or control plasmids. Images were obtained under a confocal microscope (Carl Zeiss). LeicaSP8 laser confocal microscopy was used to take representative images. autophagy related 5 (ATG5)-specific siRNA, SNCA-specific siRNA, phosphatidylinositol 3-kinase (PI3K)-specific siRNA, and their control siRNA were purchased from GenePharma. A 12-well culture plate was seeded with SNCA-overexpressing cells at 60 to 70% confluence before siRNA transfection. The cells were then carefully transfected with specific siRNA using Lipofectamine 3000 reagent (Invitrogen) following the manufacturer's instructions.

### Stimulation with Lipopolysaccharide

To mimic the inflammatory conditions of MEC, HaCaT and RHEK-1 cells were treated with LPS (Sigma-Aldrich) *in vitro*. RHEK-1 cells were seeded in six-well plates and incubated for 24 h. Next, the cells were exposed to various concentrations of LPS at 0, 100, 400, and 600 ng/ml for 24 h. We determined that 400 ng/ml was optimal for RHEK-1 cells. According to previous studies, 100 ng/ml LPS was usually used for HaCaT cells (24, 25).

### Western Blot Analysis

RIPA (radio immunoprecipitation assay) lysis buffer containing PMSF (phenylmethylsulfonyl fluoride) and protease and phosphatase inhibitors was used to lyse collected samples. Proteins were extracted by centrifugation, and the concentration was determined by a BCA assay kit (Biomed). All proteins were separated by SDS-PAGE and transferred to a polyvinylidene fluoride membrane. Next, the membrane was blocked with 5% nonfat milk, then incubated with primary and appropriate secondary antibodies. The primary antibodies and drugs used were GAPDH (5174), SNCA (A13354), interleukin (IL)-6 (A0286), IL-10 (A12255), TNF-α (A11534), sequestosome-1 (p62) (A19700), microtubule-associated proteins 1A/1B light chain 3B (LC3B) (A19665), phosphatidylinositol 4,5-bisphosphate 3-kinase catalytic subunit alpha isoform (PIK3CA) (A0265), G1/S-specific cyclin-D1 (CyclinD1) (A11022), p21 (A19094), and ATG5 (A0203) from ABclonal. ATG7 (2631), nuclear factor kappa-light-chain-enhancer of activated B cells (NF-κB) (8242), p-NF-κB (3033), p-AKT (4060), and serine/threonine kinase (AKT) (4685) were from Cell Signaling Technology. 740Y-P (PI3K signaling activator) and Chloroquine (CQ, HY-17589) were obtained from MedChem Express. Protein bands were visualized with ECL (enhanced chemiluminescence) reagents and the ChemiDoc MP imaging system (Bio-Rad), and densitometry was analyzed using ImageJ software.

### Quantitative Real-Time Polymerase Chain Reaction

Total RNA from cells was extracted using an E.Z.N.A. Total RNA kit (Omega Bio-Tek) following the manufacturer's protocol. Synthesis of cDNA was performed using a reverse transcription kit (YEASEN). The cDNA was mixed with SYBR Green Master Mix (YEASEN); the specific primers designated below were obtained from Sangon, and then the mixture was used for quantitative real-time PCR with a LightCycler 96 Detection System (Roche) under the following conditions: 95 °C for 5 min, 40 cycles of 95 °C for 10 s, and 60 °C for 30 s. The primer sequences were as follows:

SNCA-forward: AAGAGGGTGTTCTCTATGTAGGC.

SNCA-reverse: GCTCCTCCAACATTTGTCACTT.

GAPDH-forward: GGAGCGAGATCCCTCCAAAAT.

GAPDH-reverse: GGCTGTTGTCATACTTCTCATGG.

### Cell Viability and Proliferation

HaCaT cells were seeded at a density of 2 × 10^3^ cells per well in 96-well plates, and RHEK-1 cells were seeded at a density of 800 per well, and all cells were incubated at 37 °C and 5% CO_2_ for 24 h. Next, the cells were exposed to corresponding LPS for 24 h. The cells were then incubated in 3-[4, 5-dime-thylthiazol-2-yl]-2, 5-diphenyl tetrazolium bromide (MTT) solution (5 mg/ml) for 4 h. After dimethyl sulfoxide was added to each well, absorbance was measured with a microplate reader at 570 nm.

Next, flow cytometry was used to assess 5-ethynyl-2′-deoxyuridine (EdU)-positive replicating cells. In brief, 10 μM EdU (Beyotime) was added to each well and incubated for 24 h at room temperature. Next, the wells were fixed with 4% paraformaldehyde (PFA) for 30 min and permeabilized in 0.5% Triton X-100 (Sigma, Germany) for 20 min at room temperature. A reaction cocktail containing sulfo-Cy5-azide, CuSO4, and ascorbic acid in PBS was used to label and detect EdU in cells. The fluorescence of EdU was detected *via* a flow cytometer (BD Bioscience, FACS Canto TM II). Flow Jo software was used to analyze DNA replication using histograms of Edu ± cells. For colony formation assays, the transfected cells (1000 cells for HaCaT; 500 cells for RHEK-1) were seeded into 12-well plates and cultured in an LPS-containing medium for approximately 2 weeks. Next, cells were rinsed with PBS, fixed with 4% PFA for 30 min, and stained with crystal violet at room temperature. The stained colonies were photographed and counted.

### Wound Healing and Transwell Assay

HaCaT and RHEK-1 cells were transfected with SNCA-overexpression and control vectors for wound healing and transwell assays. Cells were plated in six-well plates (3 × 10^5^), and scraped a straight line with the tip of a 10-μl pipette. After 24 h of incubation at 37 °C, a serum-free medium containing the same concentration of LPS was added (RHEK-1 cells with 2% FBS). The relative migration distance was measured under 10× magnification by taking photos at indicated times. For the transwell assay, the transfected cells (4 × 10^5^ cells for HaCaT; 1 × 10^5^ cells for RHEK-1) were cultured in 0.2 ml LPS-containing serum-free medium in the upper chamber and 0.6 ml medium containing 20% FBS in the lower chamber. Cells were fixed with a 4% PFA solution and stained with crystal violet after incubation at 37 °C and 5% CO_2_ for 48 h. Cotton swabs were used to remove nonmigratory cells in the upper chamber. Cells were photographed and counted in five random fields under an inverted microscope.

### Immunofluorescence Microscopy

We fixed cells with 4% PFA for 30 min, permeabilized them with 0.5% Triton X-100, and blocked them with 3% bovine serum albumin. Next, cells were incubated with primary antibodies overnight at 4 °C. Then, the cells were washed 3 times with PBST, incubated with diluted Alexa Fluor 488 goat anti-mouse IgG secondary antibodies (Cell Signaling Technology) for 1 h, then incubated in DAPI for 15 min. All images were acquired using a Thermo Scientific Cellomics ArrayScan VTI.

### Statistical Analysis

All experiments were repeated thrice. The values were expressed as the mean ± SD. Statistical differences were determined by the Student's *t* test or one-way analysis of variance (ANOVA) using GraphPad Prism 7.0 software. *p* < 0.05 was considered statistically significant. The following designations were used in the figures: ns: *p* > 0.05; ∗*p* < 0.05; ∗∗*p* < 0.01; ∗∗∗*p* < 0.001; and ∗∗∗∗*p* < 0.0001.

## Results

### Differentially Expressed Proteins in Middle Ear Cholesteatoma and Paired Ear Auditory Canal Skin

**Fig. 1:**
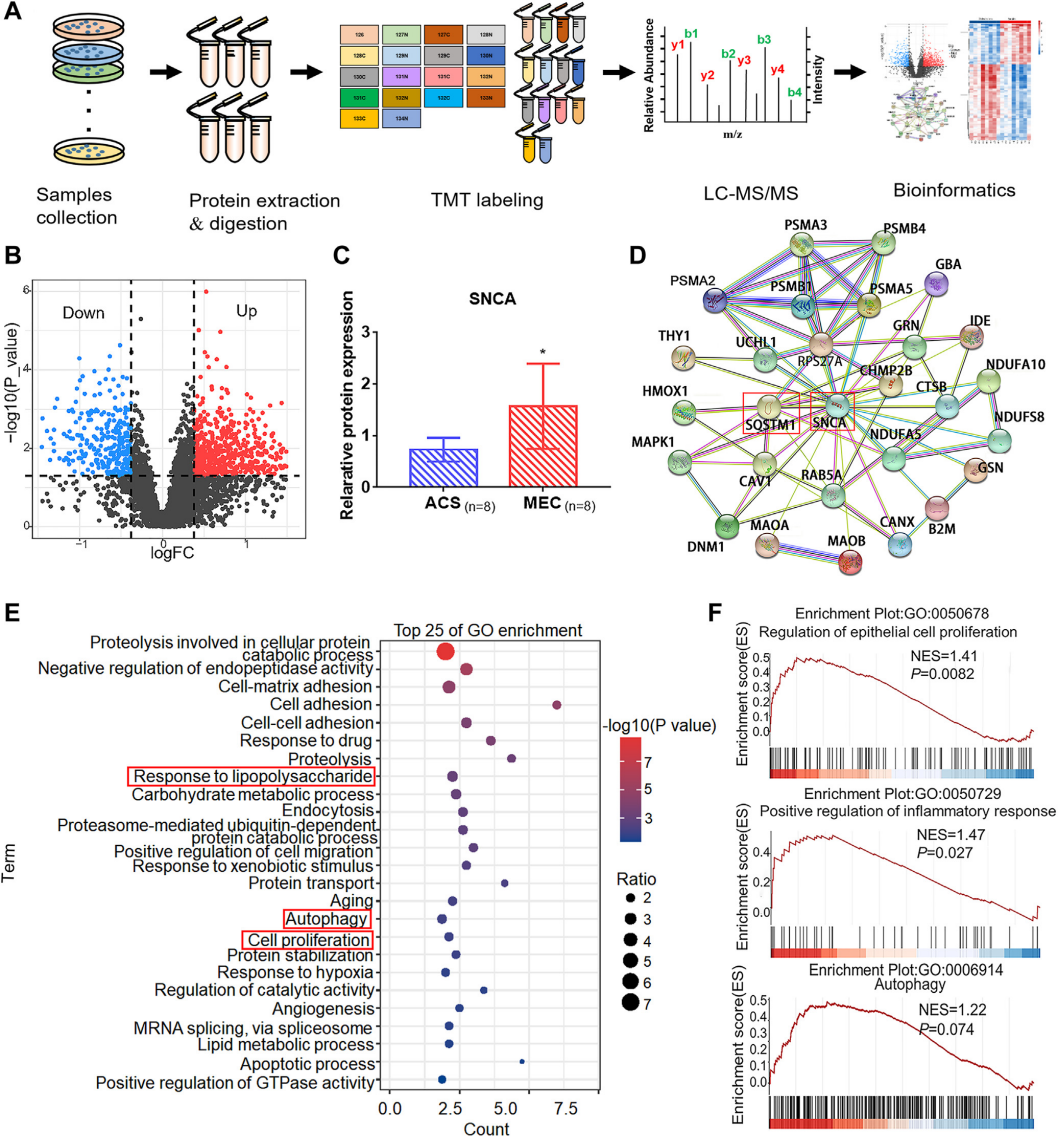
**Proteomic analysis of middle ear cholesteatoma (MEC) and auditory canal skin (ACS) to identify differentially expressed proteins (DEPs).***A*, workflow for tandem mass tag (TMT) protein profiling in this study. *B*, the volcano plot identified upregulated and downregulated proteins in MEC and ACS. *C*, the expression of SNCA in MEC was elevated compared with ACS among DEPs. *D*, protein–protein interaction network showed SNCA was closely relevant to SQSTM1 (p62). Edges represent protein–protein associations. Data were presented as mean ± SEM. ∗*p* < 0.05. *E*, the DEPs were enriched in the top 25 most significant biological process enrichment terms. The size of the spot represents the fold change corresponding to each term, and the color represents –log (*p* value). *F*, proliferation, autophagy, and inflammatory response proteins were upregulated in MEC tissues but not ACS tissues, as determined *via* gene set enrichment analysis analysis.

To identify potential molecular factors relevant to MEC, we performed proteomic analysis of eight pairs of MEC tissues and matched ACS tissues using isobaric TMT labeling. The experimental design of this study is shown in Figure 1*A*. In total, 5244 proteins (unique peptides ≥1) were identified from 37,552 unique peptides, and 5059 proteins were quantified across all 8 pairs samples (supplemental Table S1). We use a *p*-value ≤0.05 (Student’s *t* test) and the absolute value of log2 ratio ≥1 (ratio >1.3 or ratio <0.76) as the threshold to determine significance of protein expression difference. A total of 923 proteins were defined as DEPs, among which 633 were upregulated and 290 were downregulated in MEC (supplemental Table S1). The DEPs were displayed *via* a volcano plot (Fig. 1*B*). SNCA was one of the most highly expressed DEPs in MEC (Fig. 1*C*).

To screen for key proteins in MEC, the Cytohubba application in Cytoscape software was used to screen 10 hub proteins with the highest degree in DEGs, and SNCA was found to be included in them (supplemental Fig. S1*A*). To find hub proteins between DEPs, we constructed protein–protein interaction networks using STRING database and Cytoscape software. Although RPS27A, ITGB1, and CAT were top hits that are above SNAC, SNCA was a key protein that interacted with both autophagy-related and inflammation-related proteins (Fig. 1*D* and supplemental Fig. S1*B*). Our gene ontology analysis filtered multiple terms such as cell proliferation, autophagy, and response to lipopolysaccharide among the top 25 biologically enriched DEPs (Fig. 1*E*). The gene set enrichment analysis plot displayed that MECs tend to highly express genes related to epithelial cell proliferation, inflammatory response, and autophagy (Fig. 1*F*). To the best of our knowledge, inflammation is an important stimulating factor for the proliferation of cholesteatoma epithelial cells (6, 26). Furthermore, our results and recent reports have demonstrated the involvement of autophagy in the pathogenesis and progression of cholesteatoma (14). Our findings suggested MEC might be caused in part by autophagy, and SNCA might affect keratinocyte proliferation during inflammation by participating in autophagy.

### SNCA and Autophagy Markers Were Upregulated in MEC Accompanied by Autophagy Activation

**Fig. 2:**
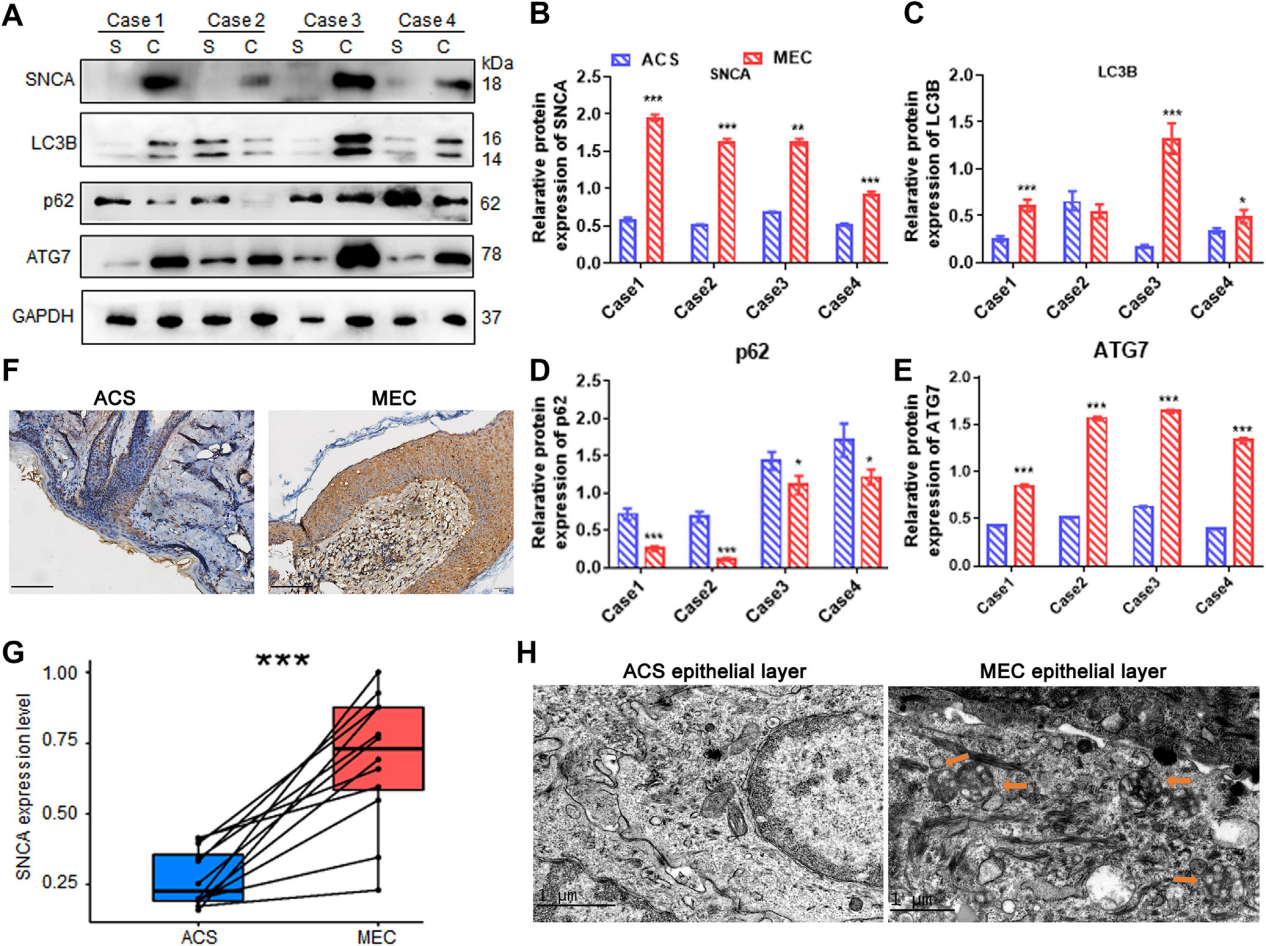
**Autophagy flux, autophagy proteins, and SNCA expression are elevated in MEC tissues.***A*, expression levels of SNCA, LC3B, p62 (SQSTM1), and ATG7 protein in four paired MEC tissues and ACS were determined by Western blotting (C, cholesteatoma; S, skin). *B–E*, column diagrams for SNCA, LC3B, p62 (SQSTM1), ATG7 protein expression levels in the four paired MEC tissues and ACS. *F*, immunohistochemistry shows SNCA expression in MEC tissues and ACS tissues. The scale bars represent 100 μm. *G*, immunohistochemistry analysis of SNCA expression in 12 paired MEC and ACS tissues. *H*, both autophagolysosomes and autophagosomes appeared in MEC tissues but not ACS tissues under transmission electron microscopy. Autophagolysosomes and autophagosomes are indicated by *arrows*. The scale bar represents 1 μm. Data were presented as mean ± SEM. n = 3. ∗*p* < 0.05, ∗∗*p* < 0.01, ∗∗∗*p* < 0.001. ACS, auditory canal skin; MEC, middle ear cholesteatoma.

Among the DEPs, we found SNCA was upregulated in MEC tissues. Fresh samples were paired to determine whether SNCA was expressed in MEC. MEC specimens showed higher SNCA expression than paired ACS specimens (Fig. 2, *A* and *B*) when analyzed by Western blot. Similarly, IHC staining showed upregulation of SNCA in MECs and its main localization occurs in cholesteatoma keratinocyte cytoplasm. Based on the stained area, integral optical density/area of SNCA protein in MEC was significantly higher than ACS (Fig. 2, *F* and *G*). The autophagy-related proteins ATG7 and LC3B-Ⅱ were also elevated, while the autophagy substrate p62 was reduced in MEC tissues (Fig. 2, *A*–*E*). We could see more autophagosomes and autophagolysosomes in MEC epithelium than in ACS epithelium under transmission electron microscopy (Fig. 2*H*). The results indicated that SNCA was overexpressed in MEC epithelium and autophagy activity was increased in MEC suggesting that SNCA might regulate autophagy in MEC epithelial cells, which must be verified through further biological investigations.

From these results we propose that the high expression of SNCA in cholesteatoma keratinocytes might promote cell proliferation by activating autophagy during inflammation. To investigate the effect of inflammation-mediated elevation of SNCA expression on autophagy induction in MEC keratinocytes and the influence of inflammation and autophagy on MEC keratinocyte proliferation, we constructed a cholesteatoma cell model with high expression of SNCA along with inflammation using cell lines of the same tissue type as MEC keratinocytes.

### SNCA-Overexpressing Keratinocytes Were More Sensitive to LPS-Induced Inflammation

**Fig. 3:**
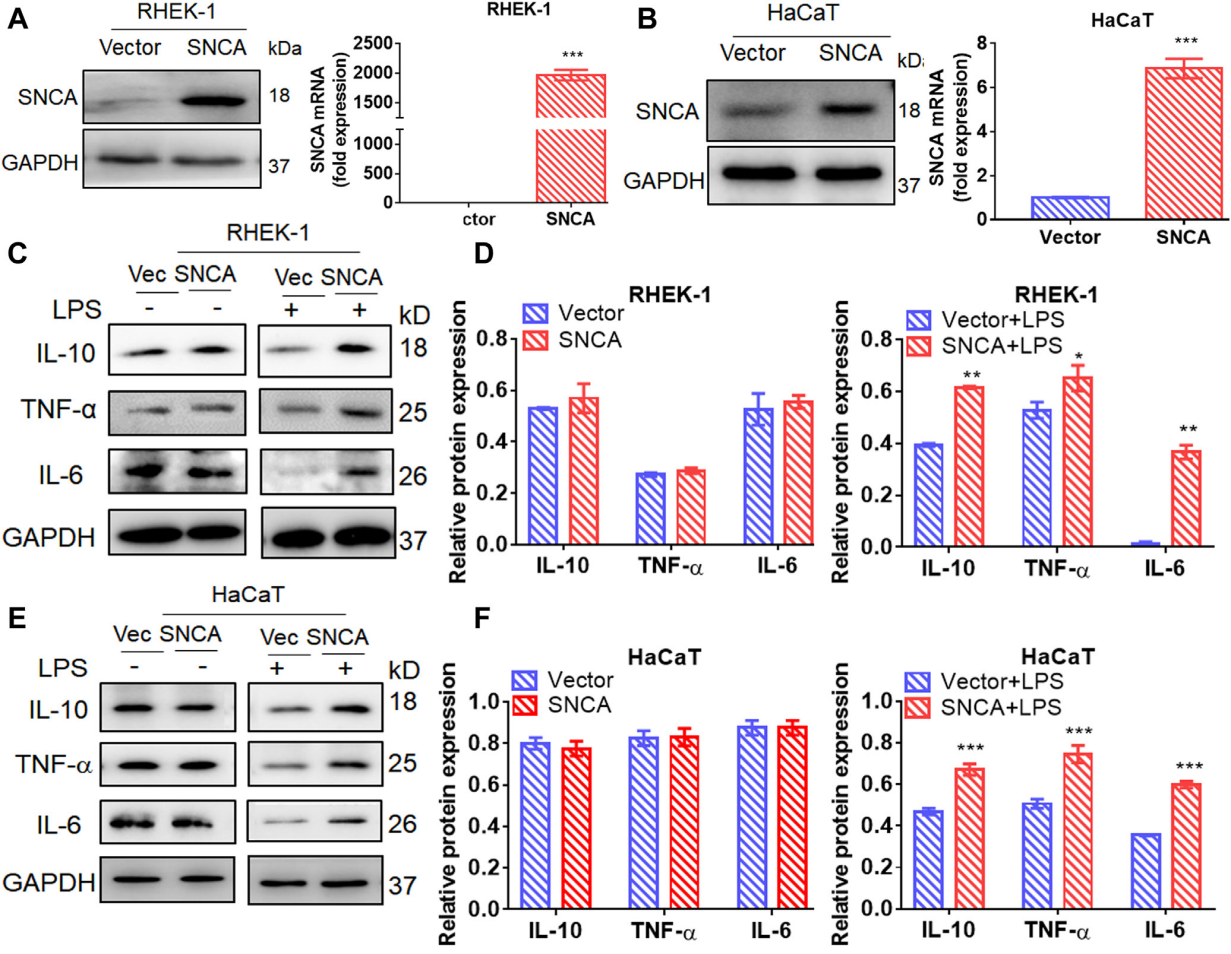
**Lipopolysaccharide (LPS)-induced inflammation was upregulated in keratinocytes overexpressing SNCA.***A* and *B*, the construct for SNCA overexpression in RHEK-1 cells and HaCaT cells. *C–F*, proinflammatory factors were induced by LPS in SNCA-overexpressing keratinocyte cell lines as detected by Western blot analysis. Quantitative analysis of relative expression density is shown in the right panel. GAPDH was used as a loading control. Data were presented as mean ± SEM. n = 3. ∗*p* < 0.05, ∗∗*p* < 0.01, ∗∗∗*p* < 0.001.

To investigate the biological function of SNCA, we used human keratinocytes HaCaT and RHEK-1 cells transfected with lentivirus stably overexpressing SNCA. The inflammation of SNCA-overexpressing keratinocytes was induced by LPS to construct a cholesteatoma cell model with high expression of SNCA. The transfected efficiency was verified by quantitative PCR and Western blotting (Fig. 3, *A* and *B*). We performed an MTT assay to determine the appropriate concentration of LPS and found that 400 ng/ml significantly increased cell viability for RHEK-1 cells and 100 ng/ml was appropriate for HaCaT cells. These concentrations were employed for subsequent experiments. SNCA-overexpressing keratinocytes were then tested for their sensitivity to inflammation induced by LPS. SNCA-overexpressing RHEK-1 and HaCaT cells treated with LPS significantly upregulated expression of IL-10, TNF-α, and IL-6 than vector-transfected control cells (Fig. 3, *C*–*F*). These results indicated that LPS effectively induced an inflammatory response in RHEK-1 and HaCaT cells, consistent with prior reports (24, 27). SNCA-overexpressing keratinocytes, however, responded more strongly to LPS.

### SNCA Contributed to Keratinocyte Proliferation and Migration Especially Under LPS Stimulation

**Fig. 4:**
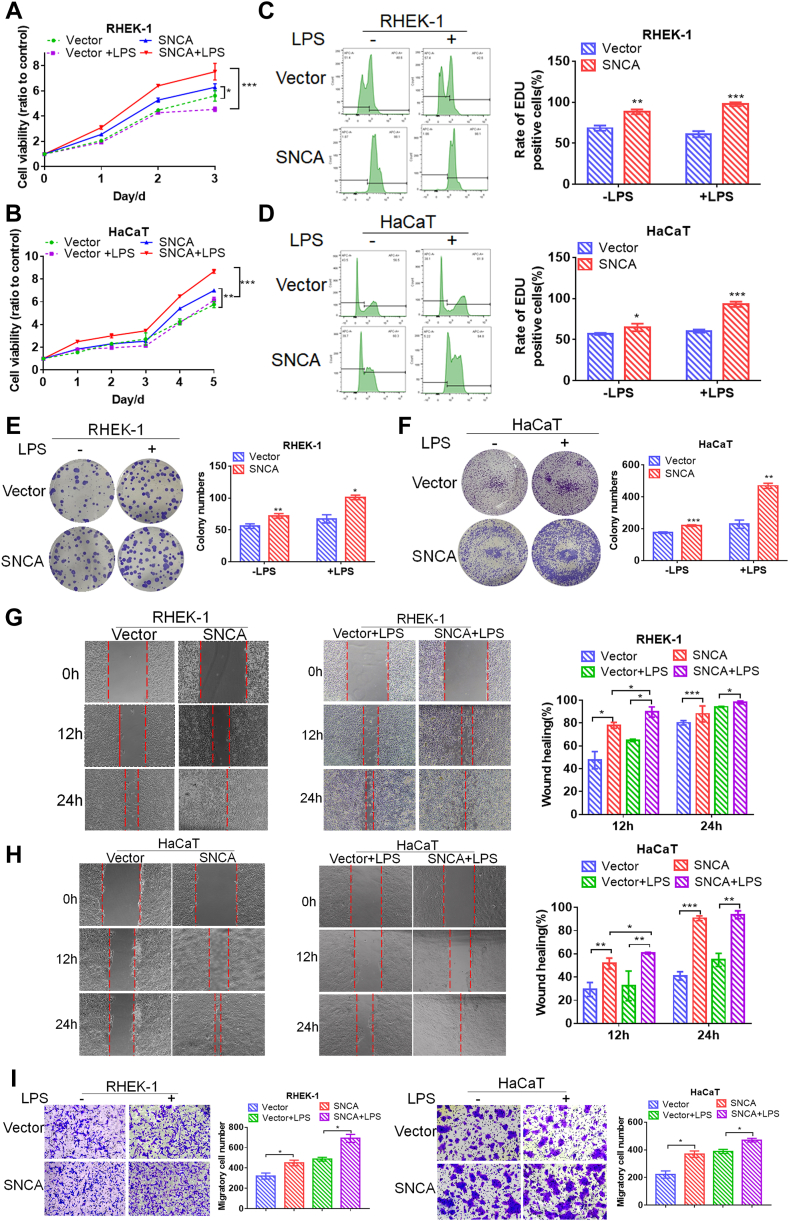
**SNCA promoted keratinocyte proliferation and migration, and lipopolysaccharide (LPS) increased this effect.** SNCA overexpression significantly promoted keratinocyte proliferation, as measured by the MTT *(A* and *B*), EdU incorporation (*C* and *D*), and colony formation (*E* and *F*) assays. The effect was more significant under LPS. Wound healing assay (*G* and *H*) and transwell assay (*I*) revealed that SNCA overexpression promoted keratinocyte migration, again more significantly under LPS. Data were presented as mean ± SEM. n = 3. ∗*p* < 0.05, ∗∗*p* < 0.01, ∗∗∗*p* < 0.001.

As a next step, we investigated whether SNCA contributes to MEC keratinocyte proliferation and migration. As shown in Figure 4, *A*–*D*, overexpression of SNCA significantly increased cell viability and EdU incorporation of HaCaT and RHEK-1 cells, which were more significantly under appropriate levels of LPS stimulation (Fig. 4). After 2 weeks culture, colony formation was higher in the SNCA-overexpressing keratinocytes than in the control cells, and it was even higher when the keratinocytes were stimulated with LPS (Fig. 4, *E* and *F*). A wound healing assay showed that HaCaT and RHEK-1 cell lines display high motility when overexpressing SNCA compared with the control group (Fig. 4, *G* and *H*). The average 24-h migration rate of SNCA-overexpressing RHEK-1 cells was nearly 100%, compared with 80.24% in the control group. Similar results were seen in HaCaT cells. The migration rate increased significantly under LPS treatment (Fig. 4, *G* and *H*). In addition, the transwell assay results were in line with the results of the wound healing assay (Fig. 4*I*). These findings indicated that SNCA overexpression promoted the proliferation and migration of keratinocytes, especially during inflammation.

### SNCA Overexpression Enhanced the Sensitivity of Keratinocyte to Autophagy Induction

Serum starvation is the most widely researched method for autophagy induction (28, 29, 30). Lipidated LC3 (LC3B-Ⅱ) levels increase transiently at phagophore initiation and decrease during autophagosome maturation. Both autophagy initiation and late-stage autophagy inhibition contribute to LC3B-Ⅱ accumulation. A lysoalkaline agent, chloroquine (CQ), inhibits the degradation of LC3B in autophagolysosomes. The difference in LC3-Ⅱ levels between cell samples treated with and without CQ represents the amount of LC3 delivered to lysosomes for degradation (*i.e.*, autophagic flux) (31). Serum starvation was used to determine the basal autophagy levels of HaCaT and RHEK-1 cells overexpressing SNCA. Under serum starvation, SNCA-overexpressing keratinocytes showed an increase in LC3B puncta compared with control cells (supplemental Fig. S2, *A*–*D*). Western blotting results showed that SNCA overexpression significantly upregulated the LC3B-Ⅱ/LC3B-Ⅰ ratio and decreased the expression of p62 (supplemental Fig. S2, *E* and *F*). GFP-LC3B punctate accumulation was more pronounced in SNCA-overexpressing keratinocytes treated with CQ (supplemental Fig. S2, *A* and *C*), suggesting that SNCA overexpression could enhance autophagy induction by serum starvation.

**Fig. 5:**
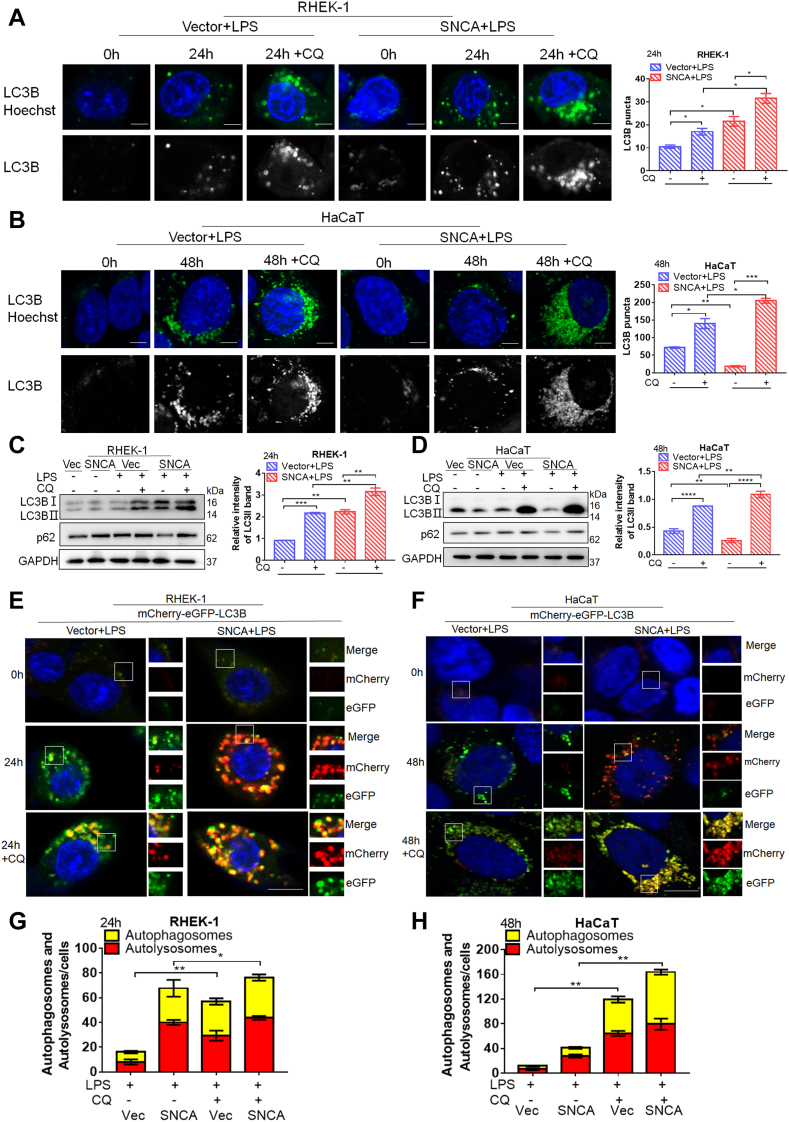
**SNCA induced significant autophagy and autophagic flux of keratinocytes stimulated by lipopolysaccharide (LPS).***A* and *B*, formation of endogenous LC3 puncta in SNCA-overexpressing RHEK-1 (*A*) and HaCaT (*B*) keratinocytes was more significant than in control cells, as detected by fluorescence microscopy. *C* and *D*, LC3-Ⅱ and p62 levels in SNCA-overexpressing RHEK-1 (*C*) and HaCaT (*D*) keratinocytes and control cells under LPS stimulation with or without 20 μM chloroquine (CQ), as detected by Western blot. *E* and *F*, autophagic flux in SNCA-overexpressing RHEK-1 (*E*) and HaCaT (*F*) keratinocytes and their vector cells in LPS stimulation with or without 20 μM CQ; the SNCA-overexpressing cells and their vector cells were infected with an mRFP-GFP-LC3 lentivirus, or cells were pretreated with 20 μM CQ. Images were then obtained by fluorescence microscopy. *G* and *H*, bar charts of LC3 punctum (*yellow* puncta for autophagosomes, red puncta for autolysosomes) quantification in SNCA-overexpressing RHEK-1 (*G*) and HaCaT (*H*) and control cells under LPS stimulation with or without 20 μM CQ. The scale bars represent 25 μm. Data were presented as mean ± SEM. n = 3, ∗*p* < 0.05, ∗∗*p* < 0.01, ∗∗∗*p* < 0.001, ∗∗∗∗ *p* ≤ 0.0001.

LPS is thought to be an effective inducer of autophagy in some cell lines (32, 33). Immunofluorescence results showed that SNCA-overexpressing RHEK-1 cells treated with LPS significantly increased endogenous LC3 puncta, while CQ treatment further increased the accumulation (Fig. 5*A*). Consistently with the Western blot assay results, LPS treatment increased LC3B-Ⅱ levels more in SNCA-overexpressing RHEK-1 cells than control cells and CQ pretreatment further increased these levels (Fig. 5*C*). Similar results were obtained from HaCaT cells. Although LC3B-Ⅱ expression and numbers of LC3 puncta significantly decreased in SNCA-overexpressing HaCaT cells compared with control cells, 48-h CQ treatment markedly increased these levels. These results indicated that LC3B was degraded more in autolysosomes of SNCA-overexpressing HaCaT cells than in control cells, resulting in higher autophagic flux in SNCA-overexpressing HaCaT cells (Fig. 5, *B* and *D*).

Because GFP fluorescence is quenched in the acidic lysosomal environment, with this probe, autophagosomes and autolysosomes are labeled with yellow (*i.e.*, mRFP and GFP) and red (*i.e.*, mRFP only) signals, respectively. As SNCA promotes autophagosome maturation, we observed that SNCA-overexpressing cells had a greater fraction of autolysosomes (red dots), suggesting SNCA increases autophagic flux. A greater accumulation of autophagosomes (yellow dots) was observed after pretreatment with CQ to inhibit autophagy flux (Fig. 5, *E*–*H*). Similar results were obtained under serum starvation (supplemental Fig. S2, *G*–*J*). These findings suggest that SNCA can promote keratinocyte autophagy and enhance autophagic flux under serum deprivation or LPS stimulation.

### SNCA Overexpression Promoted Autophagy-Mediated Cell Survival

**Fig. 6:**
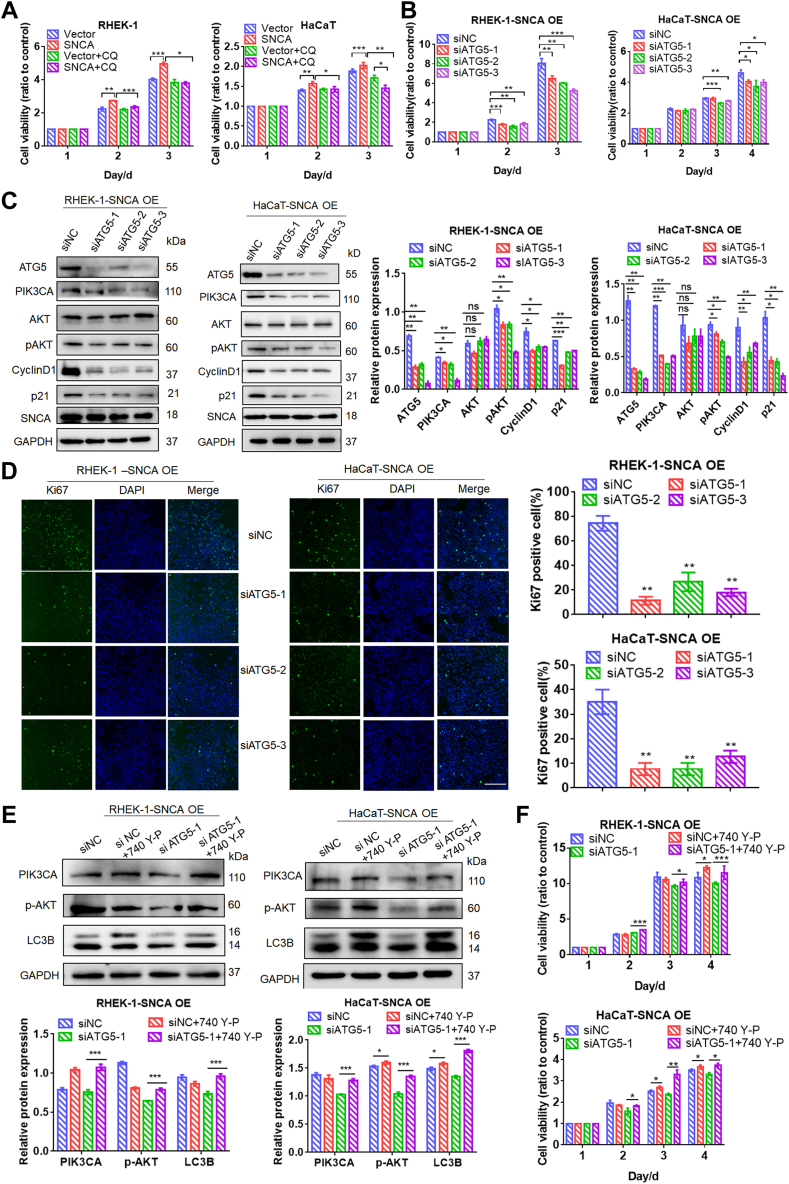
**Blocking autophagy inhibited the proliferation of the PI3K/AKT pathway in SNCA-overexpressing cells.***A*, autophagy blocker chloroquine (CQ) inhibited the proliferation of SNCA-overexpressing cells, determined the proliferation of SNCA-overexpressing cells treated with 20 nM CQ by MTT assay. *B*, silencing of ATG5 inhibited the proliferation of SNCA-overexpressing cells, determined the proliferation of SNCA-overexpressing cells transfected with ATG5 siRNA and control cells by MTT assay. *C*, silencing of ATG5 inhibited the PI3K/AKT pathway in SNCA-overexpressing cells, the protein levels of ATG5, PIK3CA, p-AKT, Cyclin D1, and p21 in the SNCA-overexpressing cells transfected with ATG5 siRNA and control cells were measured by Western blot. *D*, silencing of ATG5 inhibited the expression of Ki67 in the SNCA-overexpressing cells transfected with ATG5 siRNA and control cells, tested by immunofluorescent staining. The scale bar represents 100 μm. *E*, PI3K activator 740 Y-P reversed the inhibition of PI3K/AKT pathway and autophagy by ATG5 siRNA in SNCA-overexpressing cells, the protein levels of PIK3CA, p-AKT, and LC3B were measured by Western blot. *F*, PI3K activator 740 Y-P reversed the proliferation inhibition of ATG5 siRNA in SNCA-overexpressing cells, the proliferation of keratinocytes was detected by MTT assay. Data were presented as mean ± SEM. n = 3. ∗*p* < 0.05, ∗∗*p* < 0.01, ∗∗∗*p* < 0.001.

After confirming SNCA's association with proliferation and autophagy, we investigated the potential connection between proliferation and autophagy in SNCA overexpression conditions. The viability of keratinocytes transfected with either SNCA or vector under CQ exposure was tested with MTT assay to determine whether SNCA promotes cell survival through autophagy induction. We found that SNCA overexpression significantly increased cell viability in both HaCaT and RHEK-1 cells. However, CQ treatment reversed the boosted viability driven by autophagy in SNCA-overexpressing keratinocytes (Fig. 6*A*). To exclude off-target effects of chemical inhibitor CQ, autophagy was inhibited by silencing the autophagy essential protein ATG5 in SNCA-overexpressing keratinocytes. The results showed that the knockdown of ATG5 in SNCA-overexpressing keratinocytes decreased ATG5 protein expression and attenuated cell viability in SNCA-overexpressing HaCaT and RHEK-1 cell lines consistent with CQ treatment (Fig. 6*B*). Meanwhile, PIK3CA, p-AKT, CylinD1, and p21 expression was significantly downregulated in both SNCA-overexpressing cell lines (Fig. 6*C*). In addition, ATG5 silencing also decreased the expression of Ki67 (green) in SNCA-overexpressing keratinocytes (Fig. 6*D*). As blocking autophagy was able to decrease keratinocyte proliferation and PIK3CA expression in SNCA-overexpressed keratinocytes, we wondered if PI3K agonists could reverse this effect. As expected, PI3K agonist (740Y-P, 25 μg) reversed the autophagy block-mediated reduction in cell proliferation in SNCA-overexpressing keratinocytes (Fig. 6, *E* and *F*). These results demonstrated that autophagy inhibition reduced cell survival by suppressing PI3K/AKT/CyclinD1 signaling, supporting our hypothesis that SNCA overexpression potentially promoted autophagy-mediated keratinocyte survival through this pathway.

### SNCA Overexpression Boosted PI3K/AKT/CyclinD1 Signaling Pathway

**Fig. 7:**
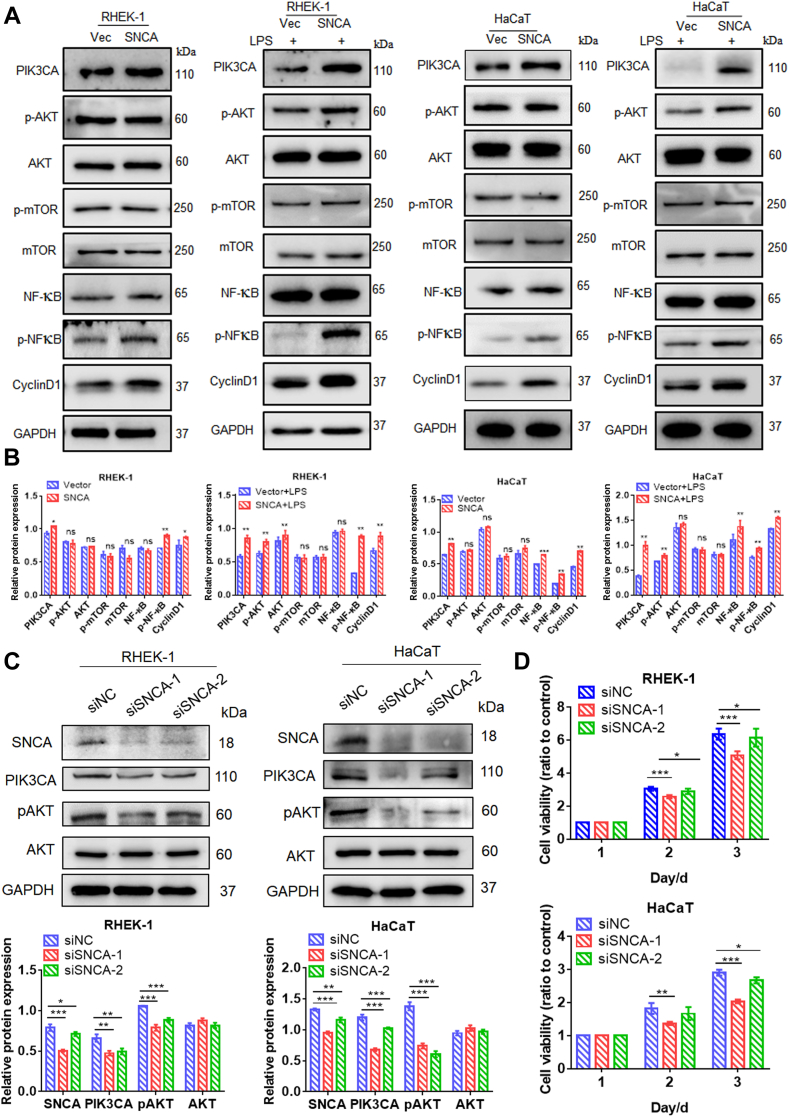
**SNCA involvement in the PI3K/AKT/CyclinD1 signaling pathway.***A*, verification of the effect of SNCA upregulation on the activation of the PI3K/AKT/CyclinD1 signaling pathway through Western blotting in RHEK-1 and HaCaT cells. *B*, quantification of relative protein expression. *C*, downregulation of SNCA expression led to decreased levels of PIK3CA and p-AKT in RHEK-1 and HaCaT cells, as determined by Western blotting. *D*, MTT assay showed that silencing of SNCA attenuated the proliferation of RHEK-1 and HaCaT cells. Data are presented as mean ± SEM. n = 3. ∗*p* < 0.05, ∗∗*p* < 0.01, ∗∗∗*p* < 0.001, ns: *p*> 0.05.

**Fig. 8:**
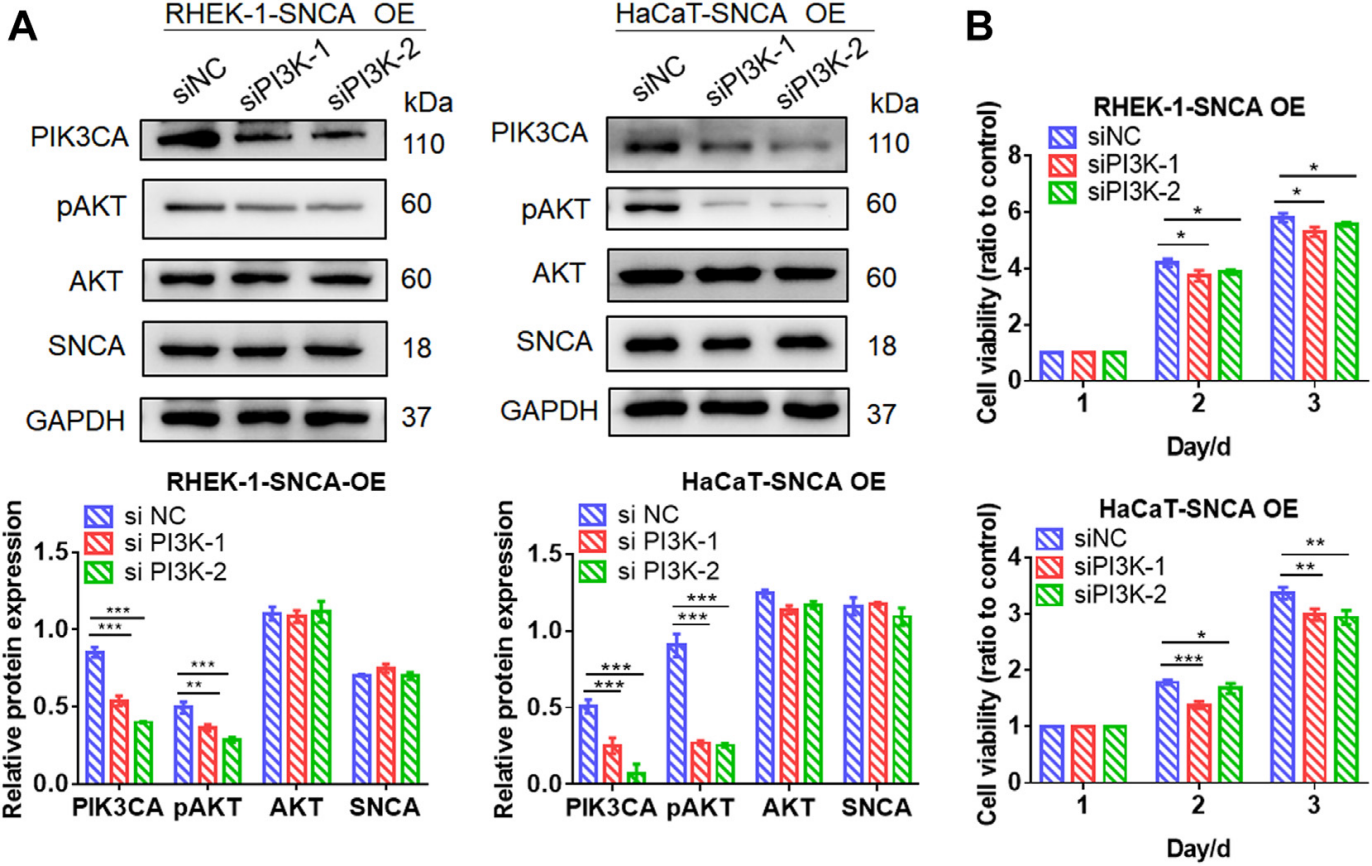
**Silencing of PI3K inhibited the proliferation in SNCA-overexpressing cells.***A*, Western blot analysis verified the knockdown of PI3K in SNCA-overexpressing cells. *B*, silencing of PI3K inhibited the proliferation of RHEK-1 and HaCaT cells, as determined by MTT assay. Data are presented as mean ± SEM. n = 3. ∗*p* < 0.05, ∗∗*p* < 0.01, ∗∗∗*p* < 0.001.

Studies have shown that SNCA was associated with regulating PI3 kinase signaling (34). The PI3K/AKT pathway was able to be aberrantly activated by various mechanisms, such as mutations in PIK3CA, PTEN, AKT, and TSC1 (35). As anticipated, PIK3CA, p-AKT, p-NF-κB/NF-κB, and CyclinD1 were increased in SNCA-overexpressing cells compared with control cells, even more significantly under LPS stimulation (Fig. 7, *A* and *B*). In contrast, knockdown of SNCA resulted in decreased protein expression of PIK3CA and p-AKT in both RHEK-1 and HaCaT cells (Fig. 7*C*) along with decreased proliferation in RHEK-1 and HaCaT cells compared with control cells (Fig. 7*D*). Furthermore, knockdown of PI3K in RHEK-1 and HaCaT cells overexpressing SNCA resulted in decreased expression of PIK3CA and p-AKT (Fig. 8*A*) as well as decreased proliferation (Fig. 8*B*).

**Fig. 9:**
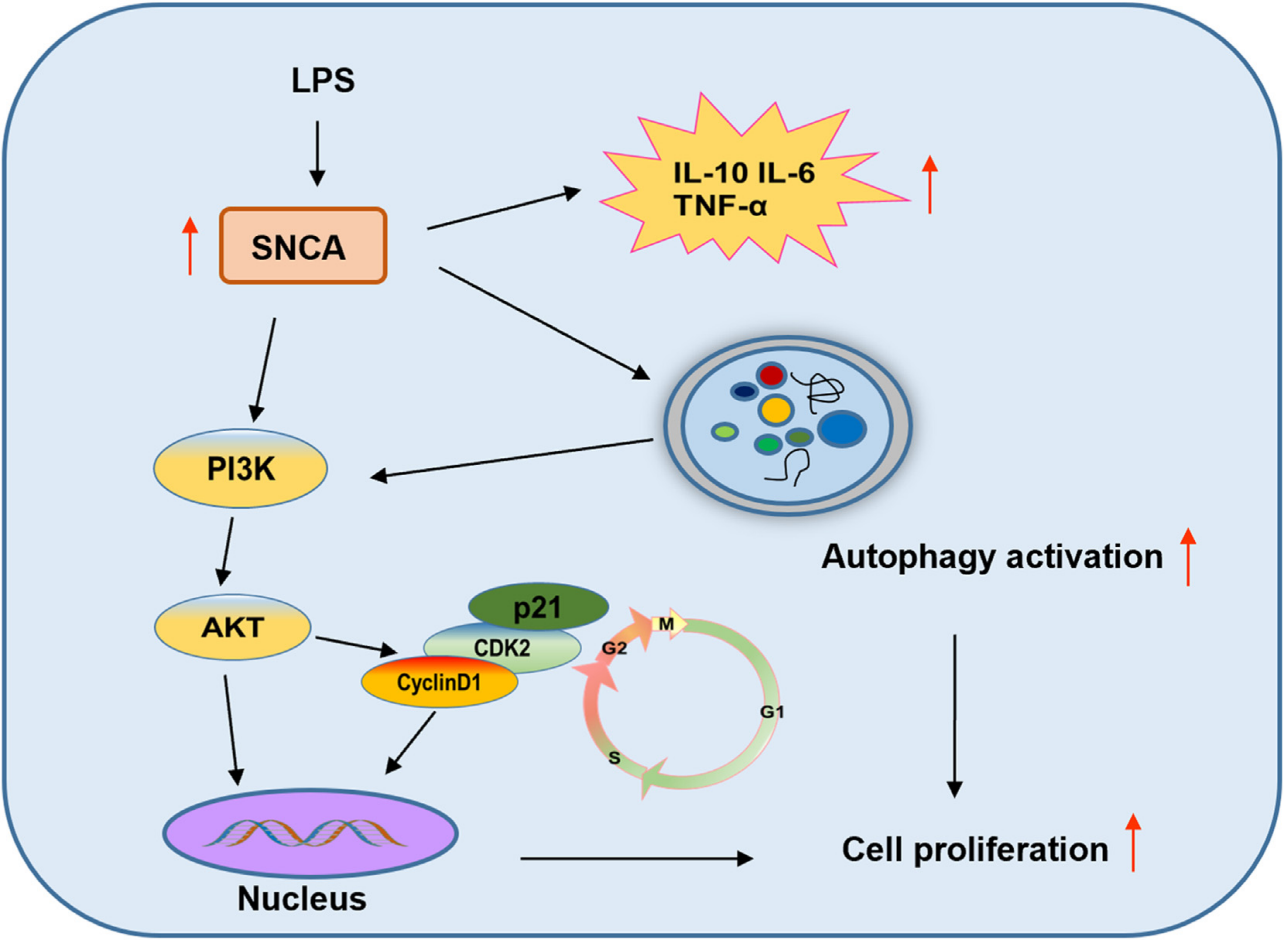
**Schematic diagram for the role of SNCA in keratinocytes.** SNCA promotes autophagy-mediated keratinocyte proliferation under lipopolysaccharide (LPS) stimulation in part *via* PI3K/AKT/CyclinD1 signaling.

In combination, our results suggest that SNCA promotes autophagy-mediated keratinocyte proliferation under LPS stimulation in part *via* PI3K/AKT signaling (Fig. 9).

## Discussion

Even though MEC is benign, it can spread to surrounding structures and cause serious, sometimes life-threatening complications (36). Proliferation, differentiation, and migration of keratinocytes under inflammatory stress are important basal phenotypes of cholesteatoma (37, 38, 39, 40). There is, however, no clear explanation for why cholesteatoma keratinocytes hyperproliferate. To investigate the molecular mechanism of proliferation and migration of cholesteatoma keratinocytes we utilized a TMT labeling high-throughput proteomics technique to directly screen and identify DEPs in MEC and paired ACS. Using a 1.3-fold change cutoff, we identified 633 upregulated proteins, including SNCA. A network analysis using bioinformatics found that SNCA was present in a cluster of important hub proteins and protein–protein interaction networks, suggesting that it is related to inflammation, proliferation, and autophagy in cholesteatoma. Furthermore, we confirmed highly expressed SNCA and autophagy-related proteins in fresh cholesteatoma tissues, as well as autophagosomes and autolysosomes, suggesting that SNCA may contribute to autophagy in an inflammatory environment, thus affecting keratinocyte proliferation. The expression and potential role of SNCA in cholesteatoma have not yet been reported.

Cholesteatoma commonly occurs in inflammatory and infectious environments. Chronic inflammation plays a crucial role in the pathogenesis of cholesteatoma (41, 42). Cholesteatoma is characterized by high levels of inflammation caused by tissue damage and infection (8). Bacterial biofilms have been confirmed in cholesteatoma. The outer layer of the bacterial bilipid membrane predominantly comprises LPS, while the inner layer consists mainly of phospholipids (42). Previous studies indicated that LPS facilitates the development and progression of cholesteatoma (38, 39, 40). Although LPS-induced upregulation of proinflammatory mediators IL-6 and TNF-α in keratinocytes have been reported (6, 27), no studies have examined the relationship between inflammation and SNCA. We observed significantly higher levels of IL-6, IL-10, and TNF-α in SNCA-overexpressing keratinocytes stimulated by LPS than in control keratinocytes, indicating greater sensitivity to LPS-induced inflammation in these cells. An SNCA-overexpressing keratinocyte model was established using LPS-stimulated SNCA-overexpressing keratinocytes, and the effects of SNCA were explored on proliferation and autophagy of keratinocytes.

Reports have indicated that SNCA is not confined to the central nervous system and its expression had considerable prognostic significance in lung adenocarcinoma (43, 44) and bladder cancer (45). The methylation of SNCA promoter was used for early detection and prognosis of Hodgkin's lymphoma (46), breast cancer (47), and colorectal cancer (48, 49). As such, SNCA may play a role in the development and progression of human cancers, with varying effects on tumor proliferation. This study confirmed that SNCA stimulates keratinocyte proliferation and migration and that the effect is more significant under inflammatory conditions. SNCA could interact with cytoskeleton components, whose rapid assembly and disassembly enable cells to migrate (50). Therefore, the alteration of SNCA (overexpression or missense mutations) could affect the microtubule system at different levels (50). The microtubule is a cytoskeleton component; it is known to play a key role in proliferative cell division in mitosis and cytokinesis (51, 52). Cell proliferation could be affected by SNCA as it regulates microtubule assembly and interacts with microtubules *in vitro* and *in vivo* (53). Thus, SNCA might have a direct or indirect effect on microtubule dynamics, thereby promoting keratinocyte proliferation, which must be confirmed by further research.

It has been reported that epidermal keratinocyte autophagy promoted keratinocyte proliferation, migration, and dermal fibroblast activation (54). Some tumor suppressor genes inhibited cancer cell proliferation by suppressing autophagy (55). Conversely, some oncogenes promote cancer cell proliferation and metastasis by triggering autophagy (56). SNCA has been shown to promote autophagy in neuronal cells (16, 17), and it serves as an autophagy-related marker in peripheral blood lymphocytes (22). However, no studies to date have investigated the effects of SNCA on autophagy in cholesteatoma keratinocytes. We hypothesized that SNCA could stimulate autophagy in cholesteatoma keratinocytes, possibly promoting proliferation.

In the present study, we found for the first time that SNCA overexpression enhanced keratinocyte sensitivity to starvation or LPS-induced autophagy. In addition, activated autophagy was also observed in cholesteatoma tissue, suggesting SNCA might be involved in autophagy processes in cholesteatoma keratinocytes. Since both autophagy and proliferation were upregulated in SNCA-overexpressing keratinocytes under inflammation, we blocked autophagy with autophagy inhibitors or by knocking down autophagy-related proteins in SNCA-overexpressing keratinocytes and assessed whether SNCA overexpression increased proliferation dependent upon autophagy activation. Prohibiting autophagy through these methods abrogated the enhanced proliferation of SNCA-overexpressing keratinocytes, demonstrating that increased proliferation due to SNCA overexpression relied on autophagy induction. Many cell cycle proteins can be modulated by autophagy, including CyclinD1. Autophagy can maintain or upregulate CyclinD1 expression to promote cell survival and proliferation in cancer cells. It is unknown whether autophagy-induced cyclinD1 promotion or maintenance is a universal mechanism for autophagy-associated cancer cell proliferation (57).

Extensive research has shown that the PI3K pathway regulates SNCA (34, 58, 59, 60). The PI3K/AKT signaling pathway exerts important effects on cell growth, proliferation, angiogenesis, apoptosis, and cytoskeletal rearrangement (61). The PI3K/AKT pathway can promote keratinocyte proliferation through the upregulation of CyclinD1 (62, 63), as CyclinD1 is a direct downstream target in the PI3K/AKT pathway (64). In the present study, we observed that PIK3CA, p-AKT, CyclinD1, and p-NF-κB expression was upregulated in RHEK-1 and HaCaT cells overexpressing SNCA. Meanwhile, inhibiting autophagy attenuated cell survival and the expression of PIK3CA, p-AKT, and CyclinD1. Interestingly, a PI3K activator (740Y-P) could reverse autophagy block-mediated inhibition of cell proliferation in SNCA-overexpressed keratinocytes, demonstrating that autophagy-mediated keratinocyte proliferation is partly mediated by PI3K/AKT/CyclinD1 signaling.

In conclusion, for the first time, we demonstrated that SNCA is overexpressed in cholesteatoma and that it may maintain cholesteatoma keratinocyte proliferation under inflammation by promoting autophagy. This may be due to SNCA promoting autophagy in cholesteatoma keratinocytes and activating the PI3K/AKT/CyclinD1 pathway, which then promotes proliferation. Therefore, SNCA and autophagy inhibition may be promising treatment strategies for cholesteatoma.

## Data Availability

The MS proteomics data have been deposited to the ProteomeXchange Consortium (http://proteomecentral.proteomexchange.org) *via* the iProX partner repository (65) with the dataset identifier PXD036926.

## Supplemental data

This article contains supplemental data.

## Conflict of interest

The authors declare no competing interests.
