# Supplementary figures and images for: The Hyperproliferation Mechanism of Cholesteatoma Based on Proteomics: SNCA Promotes Autophagy-Mediated Cell Proliferation Through the PI3K/AKT/CyclinD1 Signaling Pathway
Source: Mol Cell Proteomics. 2023 Aug 1;22(9):100628. doi: 10.1016/j.mcpro.2023.100628 (PMC10495652; doi:10.1016/j.mcpro.2023.100628)

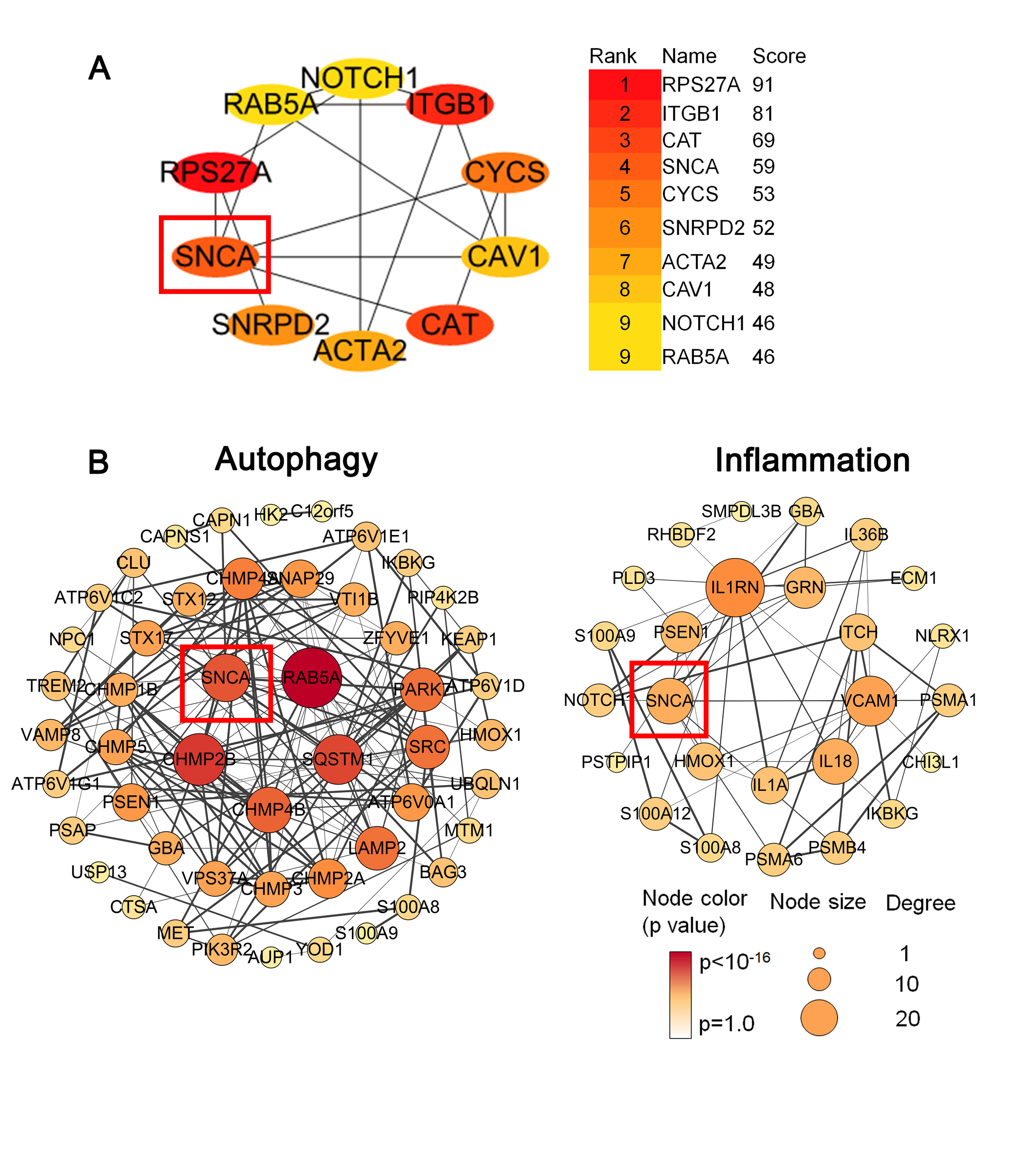

Supplement: Figure S1 [file figs1.jpg]

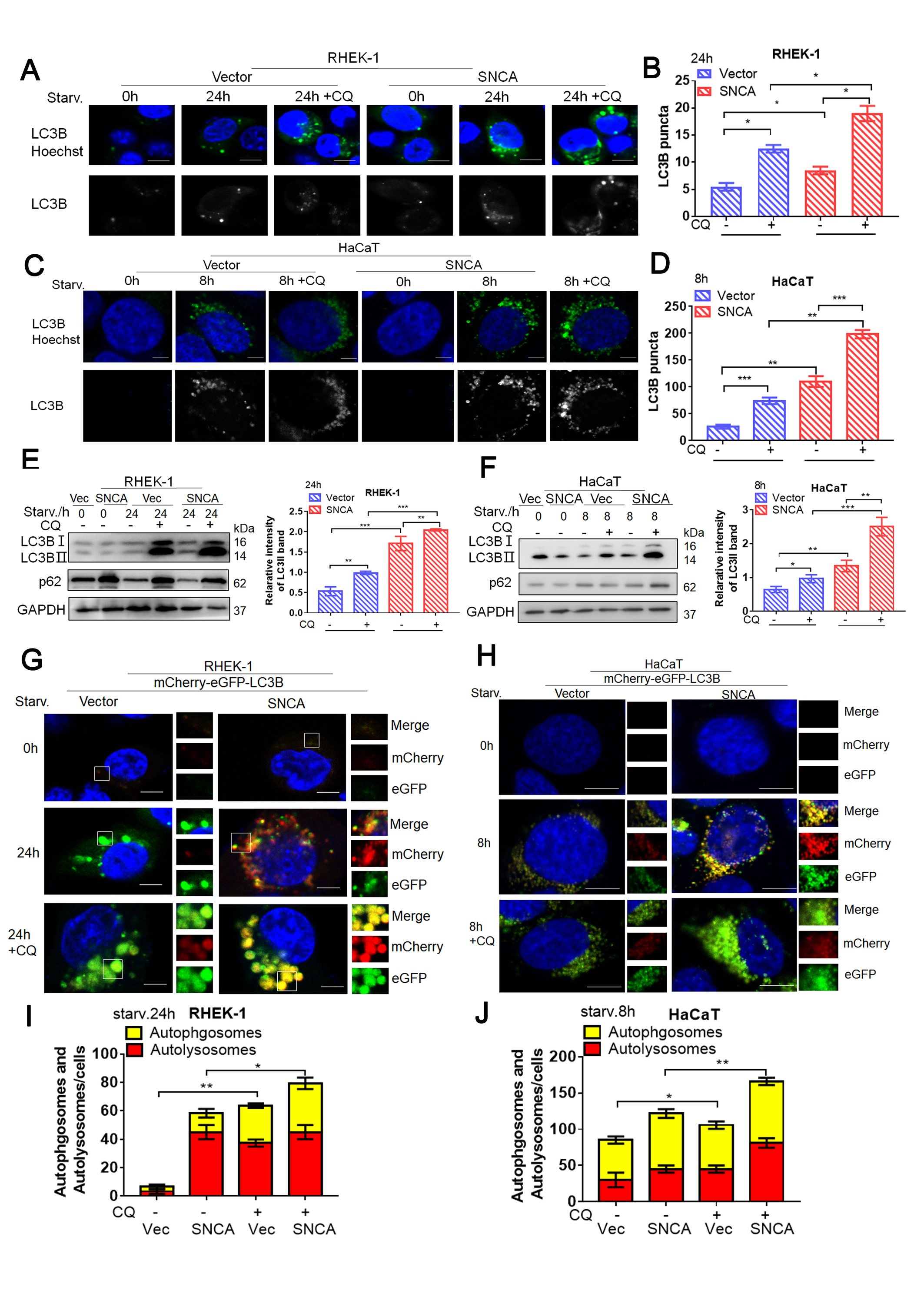

Supplement: Figure S2 [file figs2.jpg]
